# The Role of Polymers in Halide Perovskite Resistive Switching Devices

**DOI:** 10.3390/polym15051067

**Published:** 2023-02-21

**Authors:** Gregory Soon How Thien, Kah-Yoong Chan, Ab Rahman Marlinda

**Affiliations:** 1 Centre for Advanced Devices and Systems, Faculty of Engineering, Multimedia University, Persiaran Multimedia, Cyberjaya 63100, Selangor, Malaysia; 2 Nanotechnology and Catalysis Research Centre (NANOCAT), Universiti Malaya, Kuala Lumpur 50603, Malaysia

**Keywords:** polymers, halide perovskites, memory devices, resistive switching, filamentary conduction

## Abstract

Currently, halide perovskites (HPs) are gaining traction in multiple applications, such as photovoltaics and resistive switching (RS) devices. In RS devices, the high electrical conductivity, tunable bandgap, good stability, and low-cost synthesis and processing make HPs promising as active layers. Additionally, the use of polymers in improving the RS properties of lead (Pb) and Pb-free HP devices was described in several recent reports. Thus, this review explored the in-depth role of polymers in optimizing HP RS devices. In this review, the effect of polymers on the ON/OFF ratio, retention, and endurance properties was successfully investigated. The polymers were discovered to be commonly utilized as passivation layers, charge transfer enhancement, and composite materials. Hence, further HP RS improvement integrated with polymers revealed promising approaches to delivering efficient memory devices. Based on the review, detailed insights into the significance of polymers in producing high-performance RS device technology were effectively understood.

## 1. Introduction

In today’s memory storage and technology market, several types of memory that are widely used exist, including dynamic random-access memory (DRAM) [1,2] and static random-access memory (SRAM) [3,4]. Both types of memory are volatile, requiring a constant power supply to retain data. Nonetheless, there are also non-volatile memory technologies, such as flash memory, which can retain data even when the power is turned off. Flash memory is commonly used in USB and solid-state drives (SSDs) [5,6,7]. One disadvantage of flash memory is that it can only be written several times before it wears out. This process is known as “write endurance”. It can vary depending on the specific type of flash memory and how it is used. Another disadvantage of flash memory is that it is more expensive than other types of memory, such as hard disk drives (HDDs) or optical storage media, per unit of storage. Furthermore, flash memory is slower than other types of memory, such as SRAM, making it less suitable for specific applications requiring high-speed data access.

Generally, resistive memory has some advantages over flash memory, such as higher write speeds, lower power consumption, and the ability to retain data without a constant power supply (in the case of non-volatile resistive memory) [8]. Resistive memory is based on resistive switching (RS) technology, a potential candidate for future memory storage development. RS, also known as memristor behavior, is a phenomenon in which the resistance of a material changes in response to an applied electrical current [9,10]. This property has attracted significant attention recently because of its potential applications in fields such as non-volatile memory and neuromorphic computing. In a typical RS device, a thin film of material with highly conductive and resistive phases is deposited on a substrate. When a voltage is applied across the device, the current flowing through it causes the material to switch from its highly conductive phase to its resistive phase or vice versa. This change in resistance is persistent, meaning it remains even after the applied voltage is removed.

**Figure 1:**
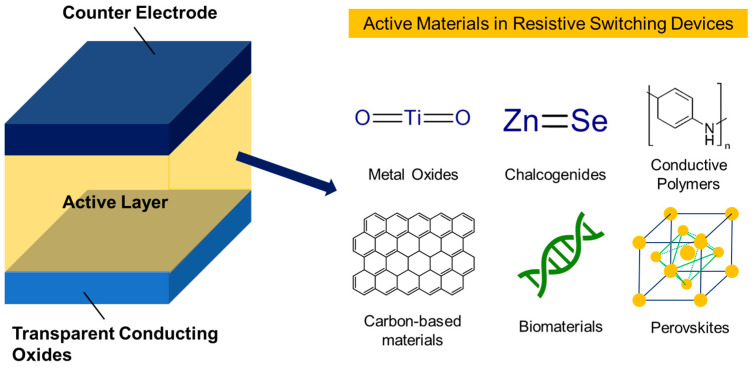
Schematic diagram of several reported active materials utilized in HP RS devices.

One of the critical advantages of RS is that it allows for the creation of non-volatile memory devices, which do not require a continuous power supply to retain information. This RS makes them highly attractive for portable and low-power electronic devices. Additionally, the ability of RS materials to exhibit both high- and low-resistance states makes them well suited for use in neuromorphic computing [11,12], where they can mimic the behavior of synapses in the brain. Currently, several types of materials are known to exhibit RS (see Figure 1), including:

1. **Metal oxides:** Metal oxides, such as titanium dioxide (TiO_2_) [13,14] and hafnium dioxide (HfO_2_) [15,16], are widely used in RS devices. When an electrical current is applied to these materials, it can cause a change in their crystal structure, resulting in a change in their resistance.
2. **Chalcogenides:** Chalcogenides are a class of materials that are composed of elements from the chalcogen group, which includes S, Se, and Te. Chalcogenides, such as Ge_2_Sb_2_Te_5_ [17] or ZnSe [18], have been shown to exhibit RS behavior when an electrical current is applied to them.
3. **Conductive polymers:** Conductive polymers are organic materials that can conduct electricity. Some examples of conductive polymers used in RS devices include polyaniline [19] and polypyrrole [20].
4. **Carbon-based materials:** Graphene [21] and carbon nanotubes [22] are widely employed in RS devices with high resistive performance. Graphene is a single layer of carbon atoms arranged in a hexagonal lattice. In contrast, carbon nanotubes are cylindrical structures made of graphene that have been rolled up into a tube shape.
5. **Biomaterials:** Biomaterials are materials derived from living organisms or their products. Hence, deoxyribonucleic acid (DNA), proteins, and enzymes are effectively proven to demonstrate RS behavior that occurs in the molecule due to the applied electrical current [23].
6. **Perovskites:** Perovskite oxides and halides are relatively new in RS devices, which makes them attractive in discovering their potential applicability. For example, researchers have demonstrated that certain perovskite, such as strontium titanate (SrTiO_3_) [24] and methylammonium lead bromide (CH3NH3PbBr_3_) [25], can exhibit RS behavior between resistance states.

Although polymers are actively researched in RS devices, halide perovskites (HPs) are alternatively explored in RS devices instead of photovoltaics. From the extensive literature on HP RS devices, only a portion includes the polymers in their design structures. These polymers act as a support to HPs to effectively improve RS properties. Furthermore, several reports described the use of polymers as having multiple functions in investigating their importance [26,27,28]. Hence, more in-depth studies should be explored to confirm the role of polymers (actively or passively) in HP RS devices as the field is gaining traction.

This review explores the role of polymers in HP-based RS devices. To the authors’ knowledge, no study has investigated the effect of polymers in optimizing RS behavior based on HPs. The influence of additional polymeric layers on ON/OFF ratios, operating voltage range, endurance, retention time, and switching mechanisms in these devices still requires further exploration. Thus, this review will aid in understanding the necessity of polymeric layers as a supporting material for high-performance HP RS devices.

## 2. HPs in RS Devices

Perovskites can be classified as halide or oxide perovskites. Halide and oxide perovskites are both materials with a particular crystal structure known as the perovskite structure. The main difference is the types of atoms that make up their crystal structure. Oxide perovskites are made up of oxygen and metals, such as Ti, Nb, or V. These perovskites are known for their high dielectric constants. They are often used in capacitors and other electronic devices. Alternatively, HPs contain a mixture of halide ions, such as Cl, Br, or I, and metals, such as Pb or Sn. HPs are often used in photovoltaic cells and LED lights due to their high efficiency and low cost. Nevertheless, RS devices employing HPs are gaining traction and will be focused on in this review.

### 2.1. Pb-Based HPs

**Figure 2:**
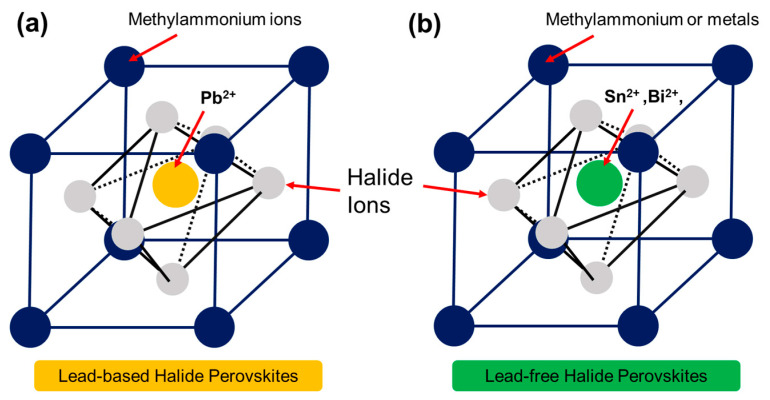
Schematic diagram of (**a**) Pb-based and (**b**) Pb-free HP structures.

Pb-based HPs are a class of inorganic materials that have attracted significant attention recently due to their unique optical and electronic properties. These materials consist of a cubic lattice of corner-sharing octahedra [29,30]. The general formula of HPs is classified as follows:

ABX_3_ (1)

where A is a monovalent cation, B is a divalent cation, and X is a halide ion (such as Cl^−^, Br^−^, or I^−^) (see Figure 2a). For example, the well-known perovskite material methylammonium Pb halide (CH_3_NH_3_PbX_3_) formula is often written as (CH_3_NH_3_)PbX_3_. This formula demonstrates that the A cation is a methylammonium ion, the B cation is a Pb ion, and the X anion is a halide ion. Hence, HPs are a subgroup of perovskites characterized by the presence of halide ions in their crystal structure.

HPs produced multiple exciting properties, such as high optical absorption, good charge-carrier mobility, and low exciton binding energy, which are helpful in various applications [31,32]. One of the critical advantages of Pb-based HPs is their tunable optical properties. By changing the composition of the material, it is possible to tune the bandgap and, thus, the material’s absorption spectrum. A study by Jahidul Islam et al. reported this alteration through the localization of halide exchange under optical trapping of CH_3_NH_3_PbBr_3_ perovskite [33]. The increase in methylammonium concentrations was able to reduce the bandgap values effectively. This observation makes Pb-based HPs highly attractive for photovoltaic devices, where they can absorb a wide range of wavelengths of light and convert them into electrical energy. In addition to their tunable optical properties, Pb-based HPs also have a high charge-carrier mobility, which allows for the efficient transport of charge carriers within the material. This property makes them well suited for use in optoelectronic devices, such as light-emitting diodes (LEDs) [34] and lasers [35].

Despite their many advantages, Pb-based HPs also have some drawbacks that must be addressed. One of the main challenges is their stability, as they are sensitive to moisture and can degrade over time. In the widely employed polycrystalline CH_3_NH_3_PbI_3_ perovskites, Alberti et al. demonstrated that Pb clusters were responsible for the degradation process when exposed to humid air [36]. Hence, researchers are currently working to improve the stability of these materials by modifying their composition or surface chemistry. Nonetheless, Pb-based HPs are promising materials with many potential applications in optoelectronic devices. Further research is needed to address their stability and other challenges, but their unique optical and electronic properties make them a promising study area.

### 2.2. Pb-Free HPs

Pb-free HPs are a class of inorganic materials that have attracted significant attention recently as an alternative to Pb-based HPs. These materials are characterized by a specific crystal structure known as the perovskite structure, which also consists of a cubic lattice of corner-sharing octahedra. Pb-free HPs are typically composed of Sn, Bi, or Ge elements in place of Pb (see Figure 2b). The critical advantage of Pb-free HPs is that they are not toxic, unlike Pb-based HPs containing Pb [37]. This advantage makes Pb-free HPs a more environmentally friendly option for applications such as photovoltaics, where the materials are in contact with the environment.

Another reason for the increasing interest in Pb-free perovskites is their potential to improve the efficiency and stability of photovoltaic cells and LED lights [38]. Some studies have shown that Pb-free perovskites have good optoelectronic properties and can achieve high efficiency under certain conditions. In addition, Pb-free perovskites are often more stable and less susceptible to degradation than Pb-based perovskites. Han et al. reported that by synthesizing Ag-doped Cs_2_NaInCl_6_ double perovskites, the stability and toxicity concerns could be avoided compared to Pb-based perovskites [39]. Overall, Pb-free HPs are a promising class of materials with many potential applications in optoelectronic devices. Thus, their sustainable optical and electronic properties make them a potential candidate for greener perovskites.

### 2.3. RS Devices

#### 2.3.1. Types of RS Devices and Mechanisms

Generally, several types of RS devices have been developed for various applications, which are as follows:

1. **Memristors:** A memristor is a type of resistor with memory, which means that it can store and retain information about the amount of current that has flowed through it. Memristors are based on the principles of resistance switching and can be used in non-volatile memory and neuromorphic computing applications.
2. **Phase change memory (PCM):** PCM is a type of non-volatile memory that relies on the phase change properties of certain materials to store data. PCM devices are based on the resistance-switching behavior of materials that can be switched between crystalline and amorphous states and can be used for high-density storage applications.
3. **Conductive-bridge RAM (CBRAM):** CBRAM is a type of non-volatile memory that relies on the resistance-switching behavior of certain materials to store data. In CBRAM, the resistance of a material is changed by the formation and breaking of a conductive filament or bridge, which an applied voltage can trigger.
4. **Resistive RAM (ReRAM):** ReRAM is a type of non-volatile memory that relies on the resistance-switching behavior of certain materials to store data. ReRAM devices can be made using various materials, including metal oxides, chalcogenides, and polymers. Due to the similarity to memristors, the topic of whether to classify as a single group today is still debated.

**Figure 3:**
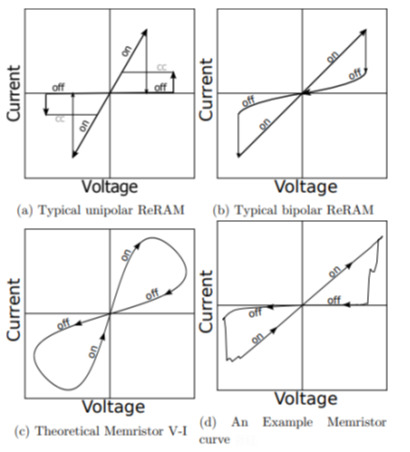
Schematic diagram of typical (**a**,**b**) unipolar and bipolar ReRAM, (**c**) theoretical memristor, and (**d**) real-life memristor curves. Reproduced with permission from reference [40].

Since the ReRAM mechanism and memristors are similar, there has always been discussion on whether they should be classified as the same thing or not [40]. Figure 3 illustrates the ReRAM and memristor producing different RS curves. The theoretical memristor demonstrated that memristor curves are often non-linear devices. Nevertheless, ReRAM’s I-V characteristic was like real-world memristors (but not 100%). According to certain researchers, such a phenomenon was caused by active memristors, conducting filaments, and non-zero crossing [40,41,42]. Bipolar or unipolar switching is evident in ReRAM curves. Therefore, ReRAM and memristor classifications continue to be contentious issues of discussion today.

**Figure 4:**
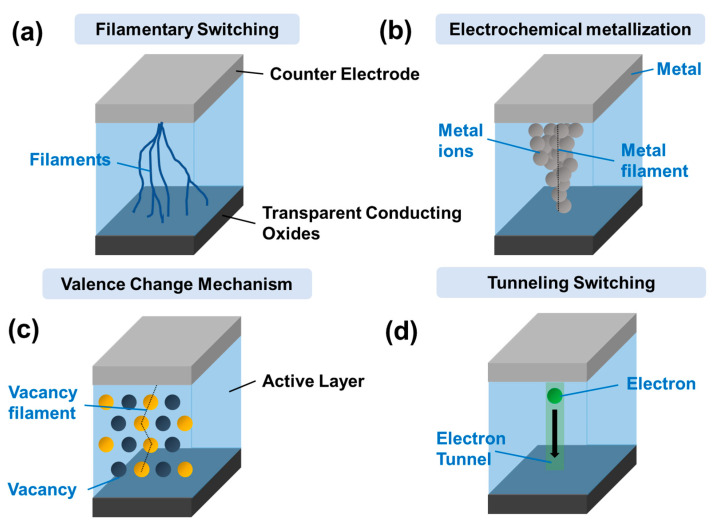
Schematic diagram of (**a**) filamentary switching, (**b**) electrochemical metallization, (**c**) valence change mechanism, and (**d**) tunneling switching of ReRAM mechanisms typically found in RS devices.

In this review, the HPs in ReRAM-based devices are studied. Several common types of ReRAM behaviors can be observed, such as unipolar and bipolar switching [43,44], multilevel [45,46], or Write-Once-Read-Many (WORM) characteristics [47]. Unipolar RS occurs when an electric current is applied to a material, and it either resists or conducts the current, depending on the direction of the applied voltage. Alternatively, bipolar RS is when an electric current is applied to a material and changes resistance in both directions when the applied voltage is changed. Multilevel RS is a type of RS that allows a material to have multiple stable resistance states, enabling it to store more information in a single device. Meanwhile, WORM RS allows the material to be written once and then read many times, but not rewritten. This type of RS is typically used in devices that require a permanent data record, such as in archival storage applications. Several types of RS mechanisms have been observed in ReRAM technology (see Figure 4). These mechanisms include:

1. **Filamentary switching:** Filamentary switching is like CBRAM, but the resistance change is caused by forming and breaking a conductive filament within the material [10,48]. This switching type is typically observed in materials such as transition metal oxides.
2. **Electrochemical metallization (ECM):** In ECM, the resistance of the material changes between a high-resistance state (HRS) and a low-resistance state (LRS) in response to an applied voltage [9,49]. The resistance change is caused by forming a metal-like phase within the material, which can be reversibly transformed into a non-metallic phase by applying a voltage.
3. **Valence change memory (VCM):** In VCM, the resistance change is caused by a change in the valence state of the material, which can be reversibly transformed by applying a voltage [50,51]. This type of switching is typically observed in materials such as chalcogenides.
4. **Tunneling switching:** This type of RS behavior occurs when an external stimulus causes electrons to tunnel through a thin insulating layer in a material, leading to a change in its resistance [52].

Overall, the different types of RS behaviors are characterized by the mechanisms underlying the resistance change. The specific type of behavior observed in a material depends on its composition and structure. Thus, understanding the specific mechanism of each fabricated device can aid in designing the addition of polymers into RS devices.

#### 2.3.2. HPs in ReRAM Devices

HPs are a class of materials studied for their potential use in ReRAM devices due to their unique properties. Although studying HPs in ReRAM devices is an active area of research, it is not a new field. In recent years, there has been increasing interest in using these materials in ReRAM devices due to their tunable electronic and optical properties, which have the potential to enable the development of new types of memory devices with improved performance and capabilities. In an ReRAM device, the resistance of the device is changed by the formation or breaking of a filament within the perovskite material, which the application of a voltage bias can induce. This change in resistance can be used to store and retrieve data in the device.

**Figure 5:**
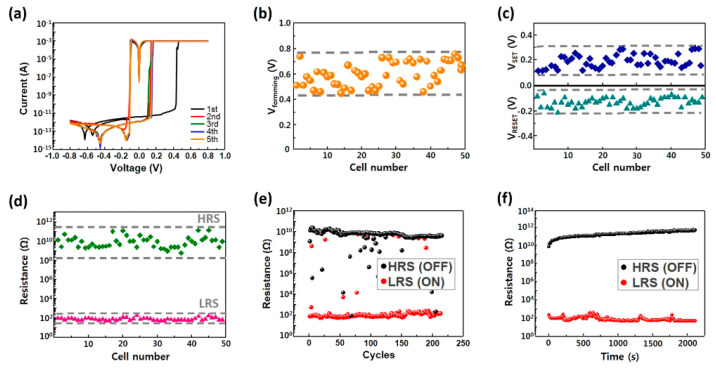
(**a**) *I*-*V* plot, (**b**) SET and RESET, (**c**) voltage distribution, (**d**) high and low resistance states, (**e**) endurance, and (**f**) retention characteristics of (PEA)_2_Cs_3_Pb_4_I_13_-based RS devices. Reproduced with permission from reference [54].

HPs can produce several properties that make them attractive for ReRAM devices. These materials can exhibit high resistance ratios (the ratio of the HRS to the LRS), which is vital for achieving an extensive dynamic range in the device. Furthermore, fast switching speeds and low power consumption are essential for high-speed and energy-efficient ReRAM devices. Recently, Kim et al. demonstrated a bipolar RS device using layered (*n*-C_4_H_9_NH_3_)_2_CsAgBiBr_7_ perovskite [53]. The study revealed excellent RS characteristics, such as an ON/OFF ratio of up to 10^7^, an endurance of 1000 cycles, and a retention time of 2 × 10^4^ s. To date, the highest ON/OFF ratio of 10^9^ was also reported by Kim et al. with quasi-2D (PEA)_2_Cs_3_Pb_4_I_13_ perovskite, owing to the uniform surface morphology (see Figure 5) [54]. Moreover, their devices were revealed to be stable under an ambient atmosphere at room temperature for five days.

In terms of endurance capabilities, a study by Siddiq et al. discovered that approximately 10^5^ cycles were largely achieved with Pb-free HPs, CsSnCl_3,_ in a flexible structure [55]. Although the ON/OFF was not high, the device revealed potential possibilities for artificial synapse studies, owing to the formation and rupturing of Ag filaments. For retention properties, Poddar et al.’s work successfully described the use of a methyl-ammonium bismuth iodide (MA_3_Bi_2_I_9_) perovskite-based ReRAM device with the highest retention time (3 × 10^5^ s) [56]. Based on the ECM mechanism, this Pb-free perovskite device produced RS behavior that opens a new pathway for ultrafast RS memories. Therefore, optimizing HP-based ReRAM devices should be the utmost priority, which can include the addition of polymers into the design structure and will be explained in the following subsection.

## 3. Influence of Polymers in HP-Based RS Devices

### 3.1. Polymers

Overall, polymers are large molecules that are composed of repeating monomer units. Monomers can be connected through covalent and chemical bonds formed when atoms share electrons [57]. Polymers can be synthesized using various methods, including chemical and electrochemical polymerization [58]. The arrangement of the monomers in a polymer can be linear, branched, or crosslinked. Linear polymers have a linear chain structure, while branched polymers have branches extending from the main chain. Crosslinked polymers have a three-dimensional network structure, with the monomers connected through covalent bonds in multiple places along the chain. The physical properties of a polymer are determined by the chemical structure of the monomers, the arrangement of the monomers, and the size and shape of the polymer molecule [59]. Some typical properties of polymers include their molecular weight, melting point, glass transition temperature, and solubility.

Polymers can be used in RS devices as active material. Several advantages exist for using polymers as the active material in RS devices. One advantage is that polymers are typically easy to process and can be synthesized in various forms, including thin films, fibers, and bulk materials. Additionally, polymers can be tailored to have a wide range of electrical and mechanical properties, making them suitable for various applications. Polymers used in RS devices include conjugated polymers, which are polymers with alternating single and double bonds in their chemical structure. Another type of polymer utilized is the conducting polymer, which can conduct electricity due to localization of conjugated π-electrons in the backbone [60]. These polymers often produce thin-film transistors and other electronic devices.

Some examples of polymers that have been explored in RS devices are polyaniline [61], polythiophene [62], polypyrrole [63], poly(3,4-ethylene dioxythiophene) [64], and poly(3-hexylthiophene) [65]. Thus, polymers are a promising material for use in RS devices due to their versatility and ability to be tailored to specific applications. Their use in these devices is an active area of research, and they will likely continue to play an essential role in developing new electronic devices. Nevertheless, this review will further explore the role of polymers as supporting materials, enhancement layers, or composite in HP RS devices.

### 3.2. Addition of Polymer Layers in Halide–Perovskite RS Devices

In this subsection, the role of polymers in HP RS devices is explored and understood. Table 1 tabulates the reported literature, including the use of polymers in HP RS devices. Polymers can be used to fabricate HP ReRAM devices in several ways. One common use is as an encapsulation layer to protect the device from the environment and prevent degradation of the HPs. Polymer encapsulation layers are used to improve the overall stability and reliability of the RS device by helping to prevent the release of harmful chemicals and reduce the risk of short circuits. Alternatively, polymers are utilized as dielectric layers in HP ReRAM devices. These layers typically insulate the device and prevent electrical current from flowing between the electrodes. Hence, polymers can form these dielectric layers due to their high dielectric constants, which can help improve the device’s overall performance. Meanwhile, polymers are used as interlayers in HP ReRAM devices to improve the adhesion between the various layers of the device and help to ensure good electrical contact between the electrodes and the perovskite material. Following this, specific types of polymers and their functions will be discussed.

#### 3.2.1. Poly(methyl methacrylate) (PMMA)

PMMA, or poly(methyl methacrylate), is a type of polymer that belongs to the family of acrylic polymers. PMMA has several properties that make it an attractive material for use in various applications, such as high tensile strength, good dimensional stability and electrical insulator, high melting temperature, transparency, and a high refractive index [77]. PMMA, or polymethyl methacrylate, is a transparent thermoplastic polymer often used to manufacture memory devices, particularly in the fabrication of interposers for 3D NAND memory devices [78]. Interposers are thin, multi-layered structures that connect memory chips to other components in a device, such as a motherboard or printed circuit board. PMMA in an RS device is typically deposited onto a substrate using a process such as spin coating or vapor deposition. The PMMA layer is then patterned using photolithography techniques to create the desired shape and size. After the PMMA layer has been patterned, it is then coated with a thin layer of a conductive material to form the top electrode.

The use of PMMA in an HP RS device is commonly reported. A study by Lee et al. described the oxide-passivated CH_3_NH_3_PbI_3_ by passivating the HP layer with SiO_2_ [67]. Thus, the multilevel bipolar switching was stable (endurance = 5.7 × 10^4^ cycles, retention = 7.8 × 10^4^ s) with the addition of PMMA, probably acting as the encapsulation layer. In Pb-free perovskites, dual-phase AgBi_2_I_7_-Cs_3_Bi2I_9_ perovskite based on ECM mechanism was successfully demonstrated by Han et al. [74]. The study effectively reported a high multilevel bipolar switching (ON/OFF ratio ≥ 10^7^) with PMMA as the supporting layer. Therefore, the PMMA was reported by the authors to act as the passivation layer while preventing HP degradation during the deposition of Au electrodes. Similarly, PMMA was used in layered (C_6_H_5_CH_2_NH_3_)_2_CuBr_4_ perovskite RS devices. The PMMA layer effectively protected the perovskite from moisture, oxygen, and reaction with Ag electrodes [75].

**Figure 6:**
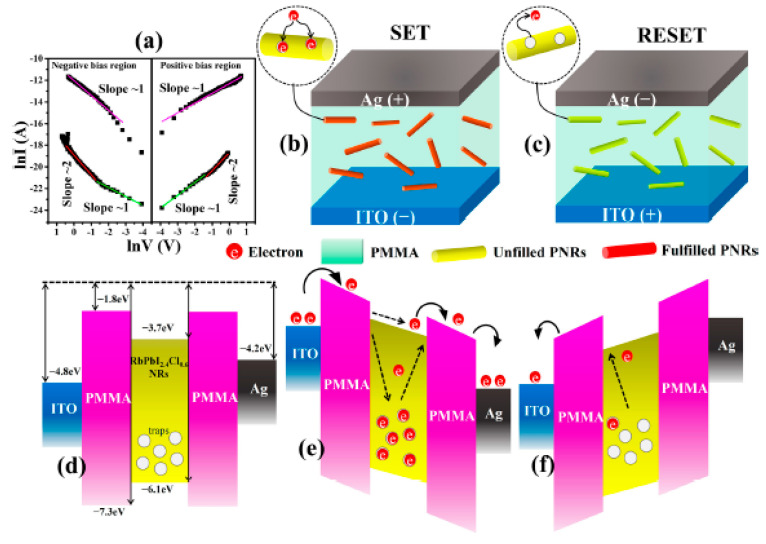
(**a**) *I*-*V* plot of 4wt%-RbPbI_2.4_Cl_0.6_@PMMA composite. (**b**) SET and (**c**) RESET process of the RS device. (**d**–**f**) Energy band diagram during the HRS to LRS to HRS transition process. Reproduced with permission from reference [70].

A few studies reported the integration of HPs within the PMMA matrices. Yang et al. demonstrated a composite layer consisting of PMMA and CH_3_NH_3_PbBr_3_ perovskite [68]. With excellent RS characteristics (ON/OFF ratio ≥ 10^3^, retention time ≥ 4000 s), the role of PMMA was reported to aid in blocking the aggregation of perovskite quantum dots. Consequently, a smooth and homogenous PMMA-CH_3_NH_3_PbBr_3_ perovskite layer was formed. PMMA was also added into an all-inorganic RbPbI_3_ perovskite as a composite material [70]. By doping the HP with varying Cl concentrations, bipolar and WORM RS behaviors were observed in the PMMA matrix at 4 and 6 wt%, respectively. Furthermore, the authors described the role of PMMA in the composite in producing sufficient barrier height to reduce charge flow, which improved RS behavior (see Figure 6d–f). Thus, the study revealed the supporting role of PPMA for efficient future RS designs.

#### 3.2.2. Poly(3,4-Ethylenedioxythiophene):Poly(Styrenesulfonate) (PEDOT:PSS)

Another commonly used polymer in HP RS devices is PEDOT:PSS (poly(3,4-ethylenedioxythiophene):poly(styrenesulfonate)), which is a type of conductive polymer that is commonly used as an electrode material in a variety of electronic devices. PEDOT:PSS is a blend of two polymers (PEDOT), which is a conductive polymer, and PSS, which is an insulating polymer. The combination of these two polymers results in a material that has good conductivity, high transparency, and good stability [79]. Thus, PEDOT:PSS is a good material for RS devices as it has excellent conductive properties and can withstand high temperatures. It is also relatively easy to process and can be deposited onto a substrate using various techniques, such as spin coating, vapor deposition, or inkjet printing [80].

In Cs-based HPs, Chen et al. demonstrated bipolar RS devices using water-soluble Cs_4_PbBr_6_ perovskite [26]. Based on VCM, the study described excellent endurance (100 cycles) with a retention time of 10^4^ s. Conversely, the authors did not mention the role of PEDOT:PSS, which was assumed to act as a conductive layer between the ITO and Cs_4_PbBr_6_ perovskite instead of as the passivation layer. CsPbBr_3_ perovskite was also reported by Liu et al. based on a flexible Al/CsPbBr_3_/PEDOT:PSS/ITO/PET structure [27]. In this study, the bipolar device produced an ON/OFF ratio of ~10^2^, 50 cycles, and 100 bending cycles. The authors also successfully integrated PEDOT:PSS into the design structure to improve the charge transfer process.

**Figure 7:**
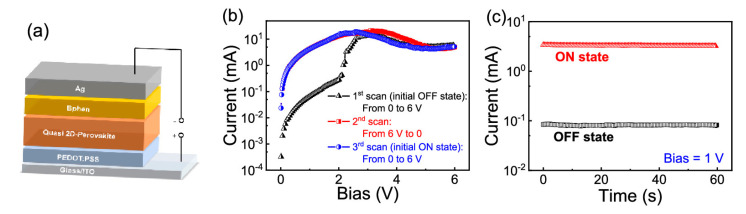
(**a**) Schematic device structure, (**b**) *I*-*V*, and (**c**) stability plots of Ag/Bphen/CH_3_NH_3_PbBr_3_/PEDOT:PSS/ITO symmetric unipolar device. Reproduced with permission from reference [28].

Pb-free perovskites utilizing PEDOT:PSS were also reported with an Al/PCBM/Cs_3_Sb_2_I_9_/PEDOT:PSS:ITO structure [76]. This highest-reported electroforming-free RS device produced remarkable ReRAM properties (ON/OFF ratio = 10^4^, retention = 10^4^ s) using layered Cs_3_Sb_2_I_9_ perovskite. The role of PEDOT:PSS was defined in this study as the hole transport layer, which effectively improved the RS characteristics of the Pb-free RS device. Yu et al. also reported unipolar RS characteristics with their Ag/Bphen/CH_3_NH_3_PbBr_3_/PEDOT:PSS/ITO symmetric device [28]. Based on a quasi-2D CH_3_NH_3_PbBr_3_ perovskite, they can achieve an ON/OFF ratio of 80 and 40 cycles, and 600 s retention time (see Figure 7b,c). Surprisingly, the authors compared this unipolar device with ITO/CH_3_NH_3_PbBr_3_/Ag, ITO/Bphen/Ag, and ITO/PEDOT:PSS/Ag, which only produced Ohmic behaviors. Similarly, this study utilizes PEDOT:PSS on the conducting substrates to increase charge transfer. Based on this subsection, PEDOT:PSS further confirmed its role as a charge transfer catalyst in HP RS devices.

#### 3.2.3. Other Rare Polymers

Although PMMA and PEDOT:PSS were frequently observed in HP ReRAM devices, other rarely reported polymers were also discovered. The use of PEI in CH_3_NH_3_PbBr_3_ as an active blend was successfully demonstrated by Ercan et al. in an Al/CH_3_NH_3_PbBr_3_:PEO/Al structure [73]. WORM behavior was observed by varying the perovskite amount in the CH_3_NH_3_PbBr_3_:PEO composite with an ON/OFF ratio of 10^4^ and 10^4^ s retention time. Intriguingly, PEO has several roles in this study, which acts as a chelating agent to coordinate with PbBr_2_/CH_3_NH_3_PbBr_3_, as protection from degradation factors, and as a matrix function. Meanwhile, PEI was reported in an ITO/PEI/CH_3_NH_3_PbI_3_/PEI/metal RS device [71]. Although the ON/OFF ratio produced was smaller at 20, the endurance of the bipolar device was high at 4000 cycles. The role of PEI was demonstrated in this study as the blocking layer to prevent direct contact of the HP with the top and bottom electrodes. Moreover, the authors confirmed that PEI does not affect the RS behavior while increasing the stability of the device. The critical information obtained in this study is that the top PEI layer should be thin to allow the metal atoms to penetrate through this layer.

**Figure 8:**
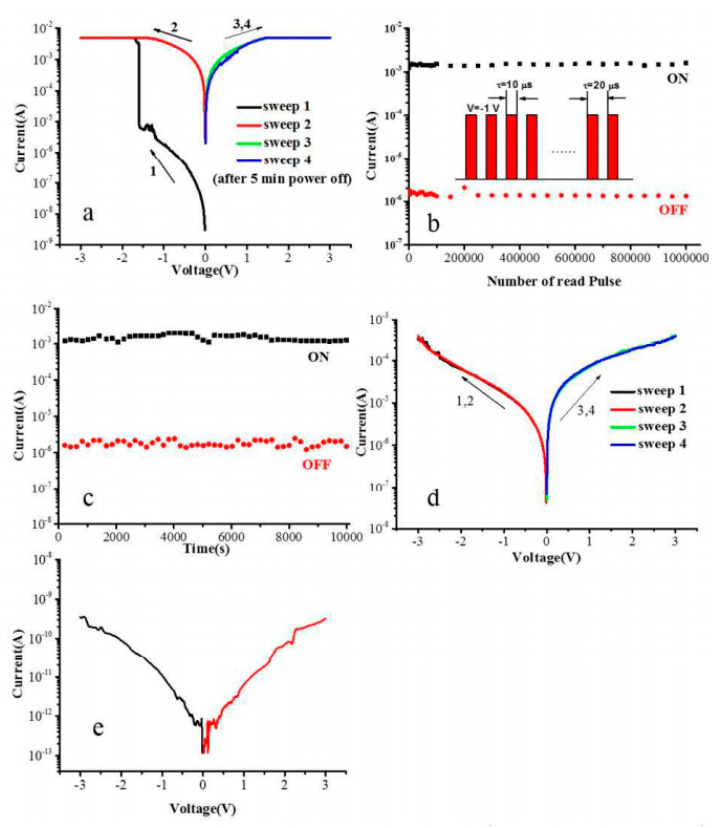
(**a**) *I*-*V* plot, (**b**) device stability under continuous read pulse, and (**c**) constant −1 V stress of ITO/CH_3_NH_3_PbI_3_:PVK/Al RS device. *I*–*V* plots of (**d**) ITO/CH_3_NH_3_PbI_3_/Al and (**e**) ITO/PVK/Al devices. Reproduced with permission from reference [72].

In another interesting study, Wang et al. discovered that the CH_3_NH_3_PbI_3_:PVK blend resulted in WORM RS behavior (see Figure 8a) [72]. When compared solely to only pure CH_3_NH_3_PbI_3_ and PVK devices, the WORM behavior diminished, thus informing the absence of RS behavior (see Figure 8d,e). Hence, the study confirmed the essential role of PVK in CH_3_NH_3_PbI_3_ perovskite in producing WORM characteristics, which were attributed to the electric-field-induced effect and conformational ordering of PVK sidechains or backbone.

## 4. Opportunities and Limitations of Polymers in HP RS Devices

### 4.1. Life Cycle Assessment (LCA)

Life cycle assessment (LCA) studies offer insights on Pb-free perovskite advantages. LCA is a method for evaluating the environmental impacts of a product or system throughout its life cycle, from raw material extraction to disposal [81]. LCA studies can be used to identify a product or system’s potential environmental impacts and identify improvement opportunities. Nevertheless, it is crucial to keep in mind that LCA has its limitations, and the results of an LCA study can depend on the assumptions and data used. When making decisions about products or systems, it is also essential to consider other factors, such as social, economic, and environmental impacts.

**Figure 9:**
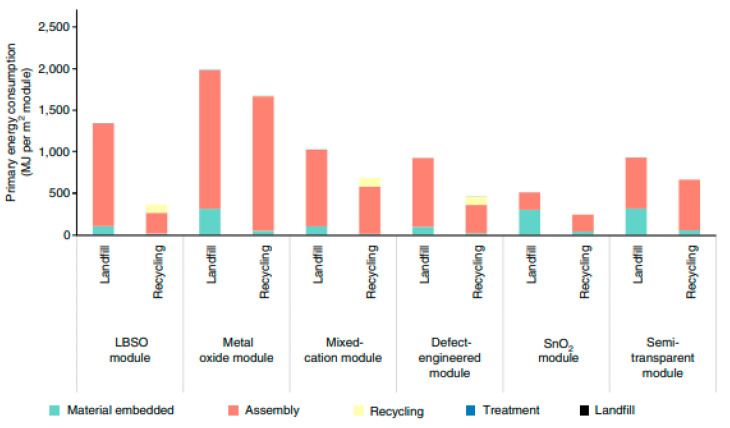
Bar chart representing the primary energy consumption between landfill and recycling of PSCs. Reproduced with permission from reference [85].

Despite several LCA reports supporting Pb-free perovskites, they are limited to perovskite solar cells (PSCs) [82,83,84]. The lack of studies on HP RS devices was nowhere to be found. Nonetheless, these PSCs LCA studies show promising strategies to combat Pb toxicity. By recycling PSC modules, a study by Tian et al. efficiently demonstrated that recycling could reduce energy payback time and greenhouse gas emissions by 72.6 and 71.2%, respectively (see Figure 9) [85]. Meanwhile, Llanos et al. concluded that Pb-free alternatives, such as mechanochemical fabricated CH_3_NH_3_SnIBr_2_ perovskite, are safer than the commonly used toxic CH_3_NH_3_PbI_3_ perovskite [86]. Therefore, similar reported LCA strategies on Pb-based and Pb-free perovskites in ReRAM devices should be urgently conducted to deliver the most efficient HP RS system. Alternatively, the role of polymers on HP RS devices has not been part of any LCA studies so far. The significance of the polymer in contributing to economic viability, toxicity, and carbon footprint in HP RS devices can offer potential design developments. Thus, further research on both materials may open new avenues in understanding their environmental impact, making material selection easier for RS devices.

### 4.2. Polymers in HP RS Devices

The study of polymeric materials in HP RS devices still requires further development to optimize these devices. Although polymers were excessively used as stand-alone polymer RS devices, their supporting role required in-depth discussions. As mentioned in Figure 8, although the author demonstrated WORM behavior in the ITO/CH_3_NH_3_PbI_3_:PVK/Al device, the amount of PVK may have affected the type of RS produced [72]. Further studies should be delivered on the polymer amount effect as it can reveal different RS behaviors. On the contrary, their pure CH_3_NH_3_PbI_3_ perovskite did not demonstrate any RS properties, which was conflictingly observed in several other reports [87,88,89]. Hence, optimization studies should be elaborated to clarify the interaction of HPs with polymers, additives, or electrodes.

Since HPs are prone to degradation from light, moisture, and oxygen, polymers were commonly utilized as passivation layers to shield the perovskite layer [90]. Only a little information was investigated on the role of polymers as additives or dopants. Nonetheless, the excessive varieties of polymers may shed some light when mixed as a composite layer. Several unexplored polymers, such as poly(vinylpolypyrrolidone) (PVP), poly(9,9-dioctylfluorenyl-2,7-diyl) end-capped with dimethylphenyl (PCDTBT), polyaniline (PANI), polypropylene carbonate (PPC), and polyethylene glycol (PEG), were still left undiscovered [91,92]. Their role in stand-alone polymer-based ReRAM devices was well established. Thus, there is potential for these polymers to be mixed with HPs. When compared to polymers in PSCs, their study was much more established. For example, Cao et al. demonstrated the use of 3D star-shaped polyhedral oligomeric silsesquioxane–poly(trifluoroethyl methacrylate)-b-poly(methyl methacrylate) (PPP) polymer in inverted PSCs [93]. Due to PPP, a superb performance was achieved (fill factor = 0.862, efficiency = 22.1%). The PPP aids in reducing defects, optimizing crystallization, and improving charge transport. Hence, they should be potentially explored in HP RS devices.

As reflected in PSCs, there is a higher potential for high-performance HP RS devices by exploring different polymers. Furthermore, the RS studies on HPs were still left behind if compared to well-established oxide and nitride RS devices. Regarding RS performance, HPs still have a long way to go to compete. Researchers should study detailed investigations to provide more opportunities and overcome the challenges of weaker resistive performance for HP RS devices.

### 4.3. Potential Design Structures

**Figure 10:**
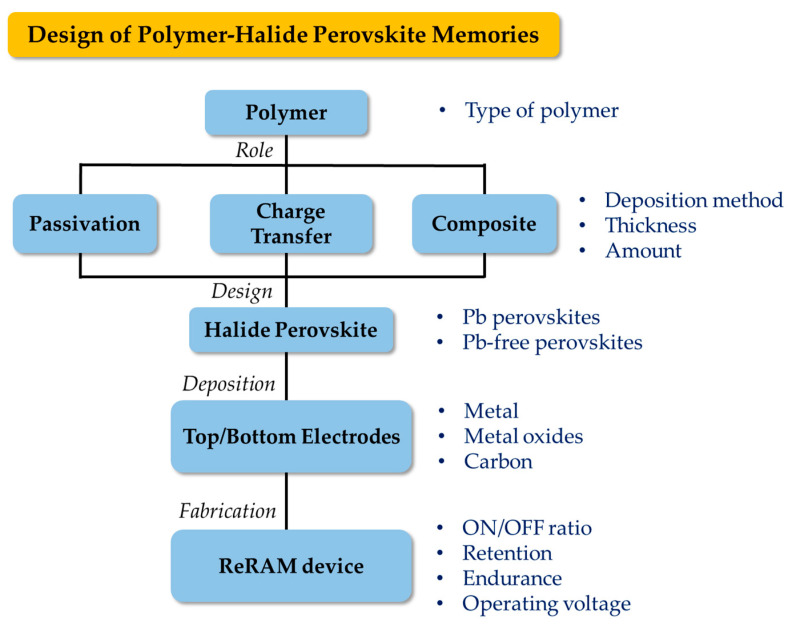
Schematic flow chart of the proposed design steps for polymer–HP RS devices.

Although reports integrating polymers in HP RS devices were not abundant, a few potential vital points can be considered when improving HP RS devices. Based on several initial studies, the following questions can be contemplated when designing the RS device structure (see Figure 10), such as:

1. What type of polymers should be selected to integrate into the ReRAM device? Since there are wide polymer varieties, multiple factors can be considered, such as suitable deposition methods, molecular weight, stability, types of polymers, and different interaction of polymers with different perovskites (Pb-based, Pb-free, Pb oxides) or electrodes (metal, metal oxides, carbon). The mass production of perovskite-based applications requires stability in addition to replacing or partially replacing a toxic element, such as Pb, with a non-toxic metal. Therefore, future research must be directed toward finding Pb-free perovskites that are stable over time and function well in RS devices.
2. What is the role of the polymer in the ReRAM device? As discussed, polymers can play multiple roles in the design structure as a passivation layer, charge transfer enhancement, matrix function, or chelating agent.
3. If a polymer is integrated as a separate layer, what is the suitable thickness? Specific RS mechanisms, such as ECM, require a thin polymer layer to allow the metal atoms to diffuse through to complete the conductive pathways by forming metal filaments. Therefore, a slight change may vary the mechanism of producing the filamentary conduction pathways.
4. If a polymer is mixed as an active composite layer, what is the appropriate wt% amount? Some reports have considered that by manipulating the blend ratio, RS characteristics can be produced from non-RS characteristics in the same blend. Thus, variation in the blend amount should be tested before confirming the non-existent RS behavior.

Although there are many factors to consider, research on the use of polymers in HP RS devices is relatively novel. Different outlooks are promising since HPs are still being initiated in RERAM technology. The use of polymers can improve the fundamental RS properties of these devices in terms of operating voltage values, power consumption, and stability. Thus, polymers may play an essential role in the electronic industry’s future development of mass-commercialized HP ReRAM-based products.

## 5. Conclusions

In conclusion, the role of polymers in HP memory devices was successfully described in this review. To date, polymers have been utilized as passivation, charge transfer, or active materials mixed with HPs. Each role would need to be carefully optimized and investigated, as certain factors, such as thickness, amount, and type of polymers, can affect the final RS output. Based on reported works, composite materials (polymers–HPs) were most promising in significantly altering the RS behaviors and memory efficiencies. Therefore, functional studies producing more new and active blends can shed some novel information on the active role of polymers in HPs. Although several recent studies have been carried out, further studies should be conducted, particularly with new polymeric materials and their interaction with HPs or the design structure. This review observed polymers as a highly versatile component in HP RS devices. Hence, this can initiate interest in understating the essential role of polymers in HP RS device technology.

## Figures and Tables

**Table 1:** Summary of reported HP RS devices utilizing polymers in their design structures.

Design Structure	Perovskite Type	Pb-Inclusion	Polymers	ON/OFF Ratio	Endurance (Cycles)	Retention (s)	Ref
Ag/PMMA/CsPbI_3_/Pt/Ti/SiO_2_/Si	CsPbI_3_	Pb	PMMA	>10^6^	300	1000	[49]
ITO/MAPbI_3_-TiO_2_/PMMA/Al	MAPbI_3_	Pb	PMMA	-	50	10^3^~10^4^	[66]
Pt/oxide-passivated MAPbI_3_/PMMA/Ag	MAPbI_3_	Pb	PMMA	10^6^	5.7 × 10^4^	7.8 × 10^4^	[67]
ITO/PMMA/MAPbBr_3_:PMMA/PMMA/Ag	MAPbBr_3_	Pb	PMMA	>10^3^	-	4000	[68]
ITO/FA-MA-Cs tri-cation perovskite/PMMA/Al	(FA_0.75_MA_0.25_)_1-x_Cs_x_PbI_3_	Pb	PMMA	>10^3^	130	10^4^	[69]
Ag/PMMA/4wt%-RbPbI_2.4_Cl_0.6_@PMMA/ITO	RbPbI_2.4_Cl_0.6_	Pb	PMMA	>10^3^	1000	10^4^	[70]
Ag/PMMA/6wt%-RbPbI_2.4_Cl_0.6_@PMMA/ITO				-	-	-	
Au/Cs_4_PbBr_6_/PEDOT:PSS/ITO	Cs_4_PbBr_6_	Pb	PEDOT:PSS	-	100	10^4^	[26]
Al/CsPbBr_3_/PEDOT:PSS/ITO/PET	CsPbBr_3_	Pb	PEDOT:PSS	~10^2^	50	-	[27]
Ag/Bphen/MAPbBr_3_/PEDOT:PSS/ITO	MAPbBr_3_	Pb	PEDOT:PSS	80	40	600	[28]
ITO/PEI/CH_3_NH_3_PbI_3_/PEI/metal	MAPbI_3_	Pb	PEI	20	4000	-	[71]
ITO/CH_3_NH_3_PbI_3_:PVK/Al	MAPbI_3_	Pb	PVK	>10^3^	-	-	[72]
Al/MAPbBr_3_:PEO/Al	MAPbBr_3_	Pb	PEO	10^4^	-	10^4^	[73]
Au/PMMA/AgBi_2_I_7_-Cs_3_Bi_2_I_9_/Pt	AgBi_2_I_7_-Cs_3_Bi_2_I_9_	Pb-free	PMMA	>10^7^	10^3^	>5 × 10^4^	[74]
Ag/PMMA/[C_6_H_5_(CH_2_)_n_NH_3_]CuBr_4_/Pt/Ti/SiO_2_/Si	[C_6_H_5_(CH_2_)_n_NH_3_]CuBr_4_	Pb-free	PMMA	~10^8^	2000	>10^2^	[75]
Al/PCBM/Cs_3_Sb_2_I_9_/PEDOT:PSS:ITO	Cs_3_Sb_2_I_9_	Pb-free	PEDOT:PSS	~10^4^	100	>10^4^	[76]

Abbreviations: MA = Methylammonium, FA = Formanidium, Bphen = Bathophenanthroline, PET = Polyethylene terephthalate, PMMA = Poly(methyl methacrylate), PEDOT:PSS = Poly(3,4-ethylenedioxythiophene) polystyrene sulfonate, PEI = Polyethyleneimine, PVK: Polyvinyl carbazole, PCBM = Phenyl-C61-butyric acid methyl ester, PEO = Poly(ethylene oxide).

## Data Availability

Not applicable.
